# EXPLORING THE ROLE OF FAMILISMO IN DIABETES SELF-MANAGEMENT AMONG LATINO OLDER ADULTS WITH DIABETES

**DOI:** 10.1093/geroni/igae098.1675

**Published:** 2024-12-31

**Authors:** Jimmy Reyes

**Affiliations:** University of Northern Iowa, Cedar Falls, Iowa, United States

## Abstract

The purpose of this study was to explore the role of familismo in diabetes self-management among first- and second-generation Latino older adults with diabetes mellitus type 2 and who reside in rural communities. In addition, this study aimed to identify how cultural beliefs and values influence daily diabetes self-management behaviors, experiences, and attitudes within the context of familismo among Latino older adults. A focused health ethnographic methodology was utilized using semi-structured interviews and participatory observation in two rural Iowa Latino communities. The 20 participants included first- and second-generation Latino older adults with diabetes mellitus type 2. The study used ethnographic data analysis methodology and 20 major codes emerged that were collapsed into three major themes. The themes were clustered around the importance of culture, the value of support systems, and the immigration experience. An important implication of this study derives from the notion that familismo is a multifaceted construct that involves protective and antagonistic elements in the management of diabetes. In addition, it is important to recognize the degree to which acculturation and generational differences may play a role in how much family and social support is needed. Researchers should explore the level and degree of familial and social support needed during initial and follow-up visits with the implementation of family involvement plans.

